# The negative association between serum albumin levels and coronary heart disease risk in adults over 45 years old: a cross-sectional survey

**DOI:** 10.1038/s41598-023-27974-w

**Published:** 2023-01-12

**Authors:** Xin-zheng Hou, En-qi Liu, Si-qi Liu, Hao Lv, Hua-feng Cui, Jing Han

**Affiliations:** 1 Department of Cardiovascular Disease, Jinan Municipal Hospital of Traditional Chinese Medicine, Jinan City, 250012 Shandong Province China; 2 College of Acupuncture and Massage, Shandong University of Traditional Chinese Medicine, Jinan City, 250355 Shandong Province China; 3 Acupuncture and Massage Rehabilitation Department, Qingdao Hospital of Traditional Chinese Medicine, Qingdao City, 266033 Shandong Province China; 4 Pediatric Orthopaedics, Affiliated Hospital of Shandong University of Traditional Chinese Medicine, Jinan City, 250014 Shandong Province China; 5 Department of Acupuncture, Affiliated Hospital of Shandong University of Traditional Chinese Medicine, Jinan City, 250014 Shandong Province China; 6 Department of Acupuncture, Affiliated Hospital of Shandong University of Traditional Chinese Medicine, 16369 Jingshi Road, Jinan City, 250014 Shandong Province China

**Keywords:** Biomarkers, Cardiology, Diseases, Risk factors

## Abstract

This study aimed to assess the correlation between serum albumin levels and coronary heart disease (CHD) risk in adults aged over 45 years. This cross-sectional study used the non-institutionalized US population from the National Health and Nutrition Examination Survey (NHANES 2011–2018) as the sample source. Multiple logistic regression was performed to evaluate the association between serum albumin levels and CHD risk. Smooth curve fitting was performed to explore potential nonlinear relationships. When nonlinear relationships were found, a recursive algorithm was used to calculate inflection points. Additionally, a piecewise logistic regression model was constructed. After adjusting for confounders, multiple logistic regression and smooth curve fitting indicated an inverse association between serum albumin levels and CHD risk [OR = 0.970, 95% CI = (0.948, 0.992)]. Subgroup analysis revealed that the negative correlation was statistically significant in the population of female patients, over 60 years, with hypertension, without diabetes. There was a correlation between serum albumin levels and CHD risk. Lower serum albumin levels were associated with a higher CHD risk.

## Introduction

Coronary heart disease (CHD) is one of the major causes of mortality worldwide, leading to approximately 1.78 million European and 0.36 million American deaths every year^1–3^. The pathological process of CHD includes coronary atherosclerosis (the main cause) and spasm. Lipid metabolism disorders, vascular endothelial cell injury, inflammation, and immune dysfunction can promote occurrence and development of coronary atherosclerosis, leading to CHD^4^. CHD is a serious disease that endanger human health, its incidence tends to be low. Therefore, identifying risk factors for CHD is of a great clinical significance^5^.

Albumin, which accounts for approximately 50% of the plasma protein, is the most abundant circulating protein in the blood. It binds to and transports various substances in the plasma and maintains blood colloidal osmotic pressure^6^. In addition, albumin has anti-inflammatory, antioxidant, anticoagulant, and antiplatelet aggregation effects^7^. These effects can inhibit formation of coronary atherosclerosis, thereby affecting occurrence and development of CHD. Therefore, the relationship between albumin levels and CHD risk has attracted increasing attention^7,8^. However, this relationship between serum albumin levels and the CHD risk has not been fully elucidated. Some studies have found that low serum albumin levels increased the incidence of cardiovascular diseases, such as myocardial infarction. However, some other studies have revealed that there was no correlation between the two^9–11^. Therefore, the relationship between serum albumin levels and CHD risk requires further study. Assessing the relationship between Serum albumin, as a biochemical index with high clinical attention for clinical intervention, and CHD risks is of great significance. On the one hand, this work can determine new factors related to CHD onset. On the other hand, it can point out the direction for follow-up basic scientific and clinical research. This might provide ideas for research on the mechanism of albumin involved in CHD onset. This study might also provide preliminary evidence-based medical assessment of whether the incidence of CHD could be affected by changes in serum albumin levels. Middle-aged and older people over 45 years of age are prone to CHD. This study aimed to assess the relationship between serum albumin levels and CHD risk in this population to determine whether there was a correlation between them.

## Materials and methods

### Study population

The study population was recruited from a cross-sectional survey—the National Health and Nutrition Examination Survey (NHANES), a large, comprehensive, and regularly updated sample of the non-hospitalized U.S. population^12^. NHANES use multistage stratified sampling to collect data every two years to determine the health and nutritional status of Americans. The database contained the demographics, physical examination, laboratory test results, diet, and questionnaires of the study population^12^.

This study collected data from 2011 to 2018, four survey cycles, and retained samples aged over 45 years, who had complete serum albumin and CHD data. The datasets used and analyzed during the current study are available from the corresponding author upon reasonable request.

According to the principle of 10 times events per variable [EPV] using multiple regression analysis, the sample size required for this study was calculated. The number of events of dependent variables with positive outcome should be at least 10 times the number of independent variables included in the multiple logistic regression analysis, to ensure that the main variables could be fully explained.

### Ethics statement

All analyses were based on data of the National Health and Nutrition Examination Survey (NHANES). And all procedures performed in studies involving human participants were in accordance with the ethical standards of the institutional and/or national research committee and with the 1964 Helsinki declaration and its later amendments or comparable ethical standards. The study was approved by the ethics review board of the National Center for Health Statistics. The detailed information located on the NHANES website.

### Variables

The independent variable of this study was serum albumin levels (g/L), and the dependent variable was CHD status (YES or NO). In addition, data on sex, age, race, family income poverty ratio (PIR), education level, marital status, body mass index (BMI), high-density lipoprotein cholesterol (HDL-C), low-density lipoprotein cholesterol (LDL-C), triglyceride (TG), aspartate aminotransferase (AST), blood creatinine, blood uric acid, white blood cell count (WBC), diabetes, hypertension, and smoking and drinking history were also collected.

Serum albumin concentration was measured using the two-color digital endpoint method by the Roche Cobas6000 (C501 module) analyzer. Regarding this test, albumin was combined with the dye bromocresol violet to form a complex. Absorbance was measures at 600 nm. The secondary wavelength was 700 nm.

CHD status was determined using a questionnaire survey. A trained interviewer asked the participants "Doctor ever told you that you had coronary heart disease?" and they answered yes or no. If they did not know or refused to answer, they were regarded as missing.

Data on sex, age, race, PIR, education level, marital status, diabetes, hypertension, smoking, and drinking history were collected from the participants by trained interviewers using a computer-assisted personal interview system. The participants were asked the following questions to determine their drinking, smoking, hypertension and diabetes status: “Was there ever a time or times in your life when you drank 4(female)/5(male) or more drinks of any kind of alcoholic beverage almost every day? By drink, I mean a 12 oz. beer, a 5 oz. glass of wine, or one and a half ounces of liquor”, “Have you smoked at least 100 cigarettes in your entire life?”, “Have you ever been told by a doctor or other health professional that you have hypertension, also called high blood pressure?”, “Have you ever been told by a doctor or health professional that you have diabetes or sugar diabetes?”. The participants answered yes or no. If they refused to answer or did not know the answer, they were defined as missing data. BMI was calculated by measuring the participants' height and weight. LDL-C, HDL-C, TG, AST, blood creatinine, and blood uric acid levels were obtained from standard biochemical profile analysis using Beckman Synchron LX20. The methods used to count white blood cells were based on the Beckman Coulter methodology of counting and sizing, in combination with an automatic diluting and mixing device for sample processing.

### Statistical analysis

Categorical variables were described as absolute numbers and constituent ratios. Continuous variables of normal distribution were described as mean ± standard deviation. Continuous variables of skewed distribution were expressed as median and quartile. The Kruskal–Wallis rank sum test (continuous variable) and Fisher exact probability test (categorical variable) were used to calculate the difference between the groups with and without CHD.

Multiple logistic regression models were used to evaluate the association between serum albumin levels and CHD risk. Serum albumin level was included in the regression model in the form of a continuous variable and classified variables. The cut-off points for converting serum albumin into categorical variables were 35, 40, and 45. When serum albumin was used as a categorical variable, dummy variable setting and trend test were performed.

We constructed three regression models based on the different confounding factors included in the analysis. Non-adjusted model unadjusted confounding factors. Adjust I model was slightly adjusted including three confounding factors: age, sex, and race. Adjust II model was completely adjusted for the following confounding factors: age, sex, race, diabetes, hypertension, HDL-C, LDL-C, and creatinine. Age, HDL-C, LDL-C, and creatinine levels were included in the regression model as continuous variables. Sex, race, diabetes, and hypertension were included as classified variables.

The screening methods for confounding factors were as follows. First, directed acyclic graphs of variables were constructed according to professional knowledge to clarify the causal relationship between variables and exclude intermediate variables. The potential confounding factors affecting independent variables or dependent variables were fitted into the fully adjusted regression model, and then excluded one by one to calculate the change in effect value. Only confounding factors with an impact greater than 10% of the effect value were retained for the final analysis. To test whether multicollinearity existed between the variables, the variance expansion factor was calculated. If the variance expansion factor of a variable was greater than 5, serious collinearity was considered and eliminated.

To evaluate the robustness of the results, we also performed a subgroup analysis to determine the association between serum albumin level and CHD risk in the participants of different ages, sex, hypertension, and diabetes groups.

In addition, smooth curve fitting and generalized additive models were used to address the non-linear relationship between serum albumin level and CHD risk. A recursive algorithm was developed for calculating the inflection point in the relationship between serum albumin level and CHD risk when non-linearity was detected. This was performed with a bi-segmented logistic regression model on either side of the inflection point to calculate the effect value.

The missing data processing methods were as follows. Samples with missing serum albumin and CHD risk data were excluded. Regarding categorical variables, missing samples were separately recorded as a group and marked as " Not recorded ". For continuous variables, normal distribution data were filled with average value, and skew distribution data were filled with median.

*P* values < 0.05 were considered statistically significant. EmpowerStats (version: 2.0. X&Y Solutions, Inc, Boston, MA. http://www.empowerstats.com) and R software, v.3.4.3 (Vienna, Austria: R Foundation for Statistical Computing, 2016 http://www.R-project.org). were used for data analysis.

## Results

### Participant selection and general characteristics

**Figure 1:**
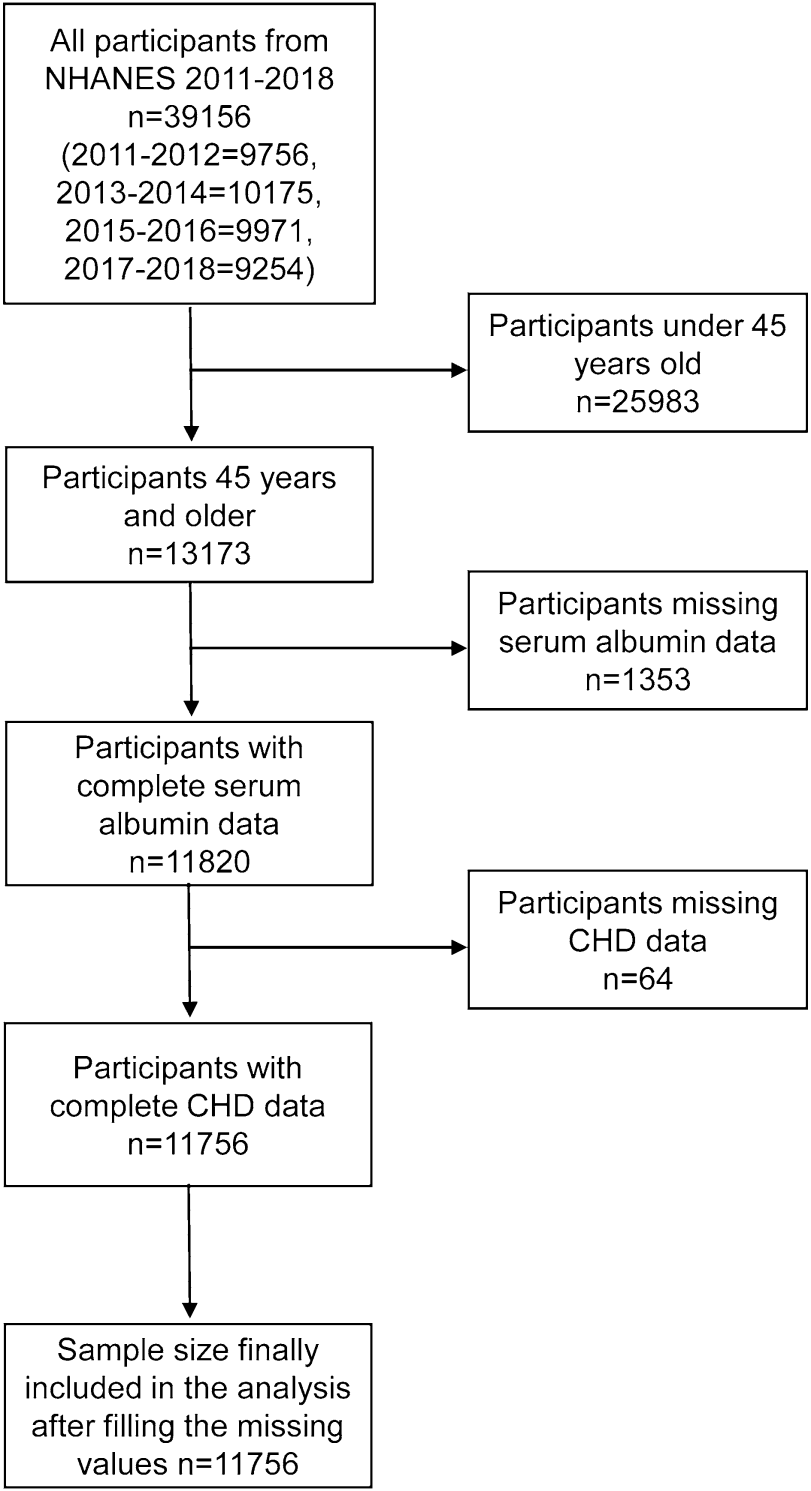
Participant screening flow chart.

A total of 39,156 participants were included in the four rounds of surveys from 2011 to 2018. After gradually excluding participants younger than 45 years and missing serum albumin and CHD data, the remaining 11,756 participants were included in the study. The sample screening process is illustrated in Fig. 1

.

Table 1 presents the study population grouped according to whether they had CHD or not. There were 799 participants with CHD, who had higher levels of age, BMI, creatinine, uric acid, white blood cell count, and the likelihood of comorbid smoking, alcohol use, high blood pressure, and diabetes. They also had lower PIR, HDL-C, LDL-C, and albumin levels.

**Table 1:** Characteristics of the study population.

CHD	No (N = 10,957)	Yes (N = 799)	*P* value
Age (year)	61.696 (10.566)	69.899 (9.200)	< 0.001
PIR	2.170 (1.240–3.970)	2.060 (1.200–3.290)	< 0.001
BMI (kg/m^2^)	29.572 (6.735)	30.015 (6.272)	0.006
HDL-C (mmol/L)	1.408 (0.435)	1.265 (0.389)	< 0.001
TG (mmol/L)	1.268 (0.775)	1.307 (0.667)	0.17
LDL-C (mmol/L)	2.959 (0.651)	2.673 (0.730)	< 0.001
Albumin (g/L)	41.714 (3.320)	41.026 (3.376)	< 0.001
AST (U/L)	22.000 (19.000–27.000)	22.000 (19.000–27.000)	0.519
Creatinine (umol/L)	76.910 (64.530–91.940)	90.170 (76.020–112.270)	< 0.001
Uric acid (umol/L)	329.151 (85.826)	357.804 (92.629)	< 0.001
WBC (1000 cells/uL)	6.800 (5.600–8.200)	7.100 (5.900–8.600)	0.003
Gender			< 0.001
Male	5197 (47.431%)	537 (67.209%)	
Female	5760 (52.569%)	262 (32.791%)	
Race			< 0.001
Mexican American	1398 (12.759%)	69 (8.636%)	
Other Hispanic	1202 (10.970%)	64 (8.010%)	
Non-Hispanic White	4225 (38.560%)	472 (59.074%)	
Non-Hispanic Black	2526 (23.054%)	114 (14.268%)	
Other Race	1606 (14.657%)	80 (10.013%)	
Education level			0.445
High school and below	5231 (47.741%)	400 (50.063%)	
Above high school	5710 (52.113%)	398 (49.812%)	
Not recorded	16 (0.146%)	1 (0.125%)	
Marital status			0.396
Living with partner	6600 (60.235%)	466 (58.323%)	
No partner	4348 (39.682%)	333 (41.677%)	
Not recorded	9 (0.082%)	0 (0.000%)	
Alcohol			< 0.001
Yes	1560 (14.237%)	159 (19.900%)	
No	7018 (64.050%)	506 (63.329%)	
Not recorded	2379 (21.712%)	134 (16.771%)	
Smoke			< 0.001
Yes	5078 (46.345%)	494 (61.827%)	
No	5869 (53.564%)	304 (38.048%)	
Not recorded	10 (0.091%)	1 (0.125%)	
Diabetes			< 0.001
Yes	2598 (23.711%)	348 (43.554%)	
No	8351 (76.216%)	451 (56.446%)	
Not recorded	8 (0.073%)	0 (0.000%)	
Hypertension			< 0.001
Yes	5502 (50.214%)	631 (78.974%)	
No	5442 (49.667%)	166 (20.776%)	
Not recorded	13 (0.119%)	2 (0.250%)	

CHD: coronary heart disease; PIR: family income poverty ratio; BMI: body mass index; HDL-C: high density lipoprotein cholesterol; TG: triglyceride; LDL-C: low density lipoprotein cholesterol; AST: aspartate aminotransferase; WBC: blood white blood cell count.

### Relationship between serum albumin and coronary heart disease

The number of participants with CHD in this study was 799 which was far more than 10 times the number of independent variables included in the multiple regression analysis. Therefore, the sample size of this study met the requirements for multiple regression analysis. The results of the regression model analysis after full adjustment for confounding factors showed that there was a negative correlation between serum albumin and CHD risk, and the results were statistically significant [OR = 0.970, 95% CI = (0.948, 0.992), Table 2]. After converting serum albumin levels to categorical variables, no negative correlation was observed [Q4 VS Q1, OR = 0.805, 95% CI = (0.497, 1.302), Table 2]. However, testing for trend was statistically significant (*P* = 0.002, Table 2).

**Table 2:** The relationship between serum albumin and coronary heart disease risk.

Exposure	Non-adjusted	Adjust I	Adjust II
Albumin (g/L)	0.942 (0.923, 0.962)	0.947 (0.926, 0.968)	0.970 (0.948, 0.992)
Albumin Q1	1	1	1
Albumin Q2	0.815 (0.534, 1.243)	0.983 (0.633, 1.528)	1.296 (0.823, 2.039)
Albumin Q3	0.627 (0.417, 0.945)	0.734 (0.478, 1.126)	1.022 (0.656, 1.592)
Albumin Q4	0.422 (0.271, 0.658)	0.523 (0.329, 0.832)	0.805 (0.497, 1.302)
P trend	< 0.001	< 0.001	0.002

Independent variable: serum albumin; dependent variable: coronary heart disease. Results are expressed as OR (95% CI).

Serum albumin grouping standard: Q1: < 35; Q2: >  = 35, < 40; Q3: >  = 40, < 45; Q4: >  = 45.

Non-adjusted model adjust for: None.

Adjust I model adjust for: Gender; Age; Race.

Adjust II model adjust for: Gender; Age; Race; Diabetes; HDL-C; Hypertension; LDL-C; Creatinine.

After stratified analysis according to sex, age, diabetes and hypertension, it was found that the negative correlation between serum albumin level and CHD risk was statistically significant in women [OR = 0.948, 95% CI = (0.911, 0.987), Table 3], the participants aged 60 and over [OR = 0.963, 95% CI = (0.940, 0.987), Table 3], participants without diabetes [OR = 0.969, 95% CI = (0.939, 0.999), Table 3] and those with hypertension [OR = 0.962, 95% CI = (0.938, 0.986), Table 3].

**Table 3:** subgroup analysis.

	Non-adjusted	Adjust I	Adjust II
Gender			
Male	0.933 (0.910, 0.957)	0.959 (0.933, 0.985)	0.978 (0.951, 1.006)
Female	0.919 (0.886, 0.953)	0.923 (0.888, 0.960)	0.948 (0.911, 0.987)
Age			
< 60	0.966 (0.915, 1.021)	0.933 (0.883, 0.986)	0.957 (0.904, 1.012)
≥ 60	0.953 (0.931, 0.976)	0.937 (0.915, 0.960)	0.963 (0.940, 0.987)
Diabetes			
Yes	0.963 (0.934, 0.993)	0.963 (0.932, 0.995)	0.972 (0.940, 1.005)
No	0.949 (0.922, 0.977)	0.955 (0.926, 0.985)	0.969 (0.939, 0.999)
Hypertension			
Yes	0.953 (0.931, 0.976)	0.943 (0.921, 0.967)	0.962 (0.938, 0.986)
No	0.967 (0.922, 1.015)	0.983 (0.934, 1.034)	0.999 (0.950, 1.052)

Non-adjusted model adjust for: None.

Adjust I model adjust for: Gender; Age; Race.

Adjust II model adjust for: Gender; Age; Race; Diabetes; HDL-C; Hypertension; LDL-C; Creatinine.

When gender, age, diabetes or hypertension is the hierarchical variable, it is not adjusted.

**Figure 2:**
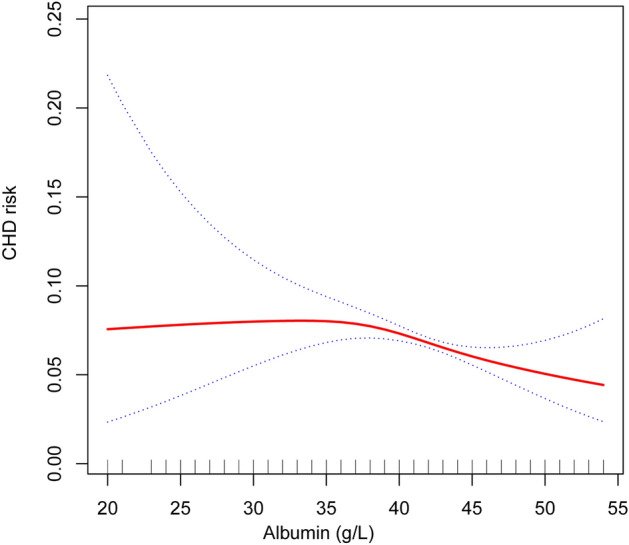
The relationship between serum albumin and coronary heart disease risk. The blue curve represents the 95% confidence interval. Adjust for: gender, age; race, diabetes, HDL-C, hypertension, LDL-C, creatinine.

Smooth curve fitting also showed a negative correlation between serum albumin level and CHD risk (Fig. 2), which appeared to have a threshold effect with a breakpoint of 36, without statistical significance (log likelihood ratio tests = 0.051, Table 4).

**Table 4:** Threshold effect.

Outcome:	CHD risk
Model I	
A straight-line effect	0.970 (0.948, 0.992)
Model II	
Fold points (K)	36
< K-segment effect 1	1.068 (0.960, 1.189)
> K-segment effect 2	0.956 (0.931, 0.982)
Effect size difference of 2 vs. 1	0.895 (0.796, 1.007)
Equation predicted values at break points	− 2.250 (− 2.401, − 2.099)
Log likelihood ratio tests	0.051

Independent variable: serum albumin; dependent variable: coronary heart disease (CHD). Results are expressed as OR (95% CI).

Adjust for: Gender; Age; Race; Diabetes; HDL-C; Hypertension; LDL-C; Creatinine.

**Figure 3:**
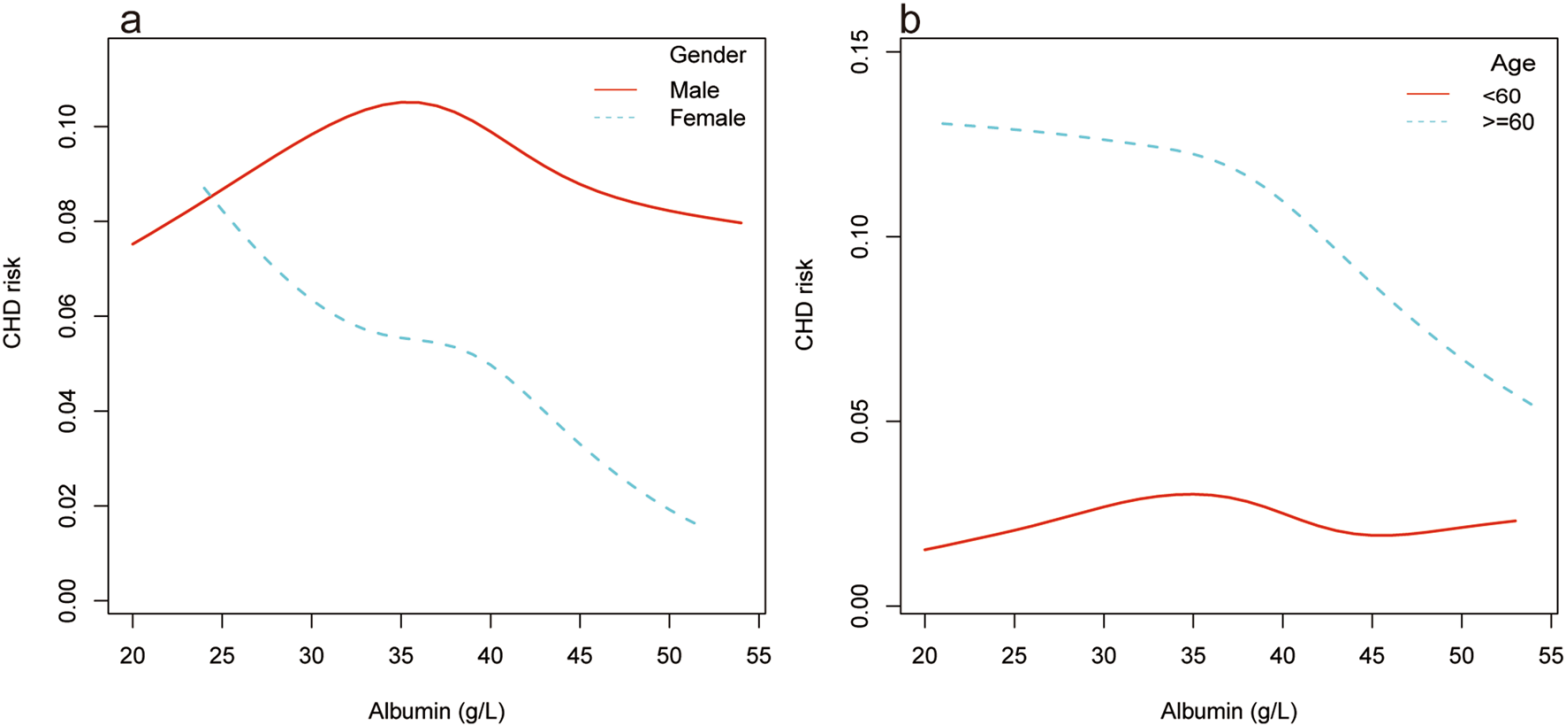
The relationship between serum albumin and coronary heart disease risk stratified by age and gender. Adjust for: gender, age; race, diabetes, HDL-C, hypertension, LDL-C, creatinine. When gender or age was the hierarchical variable, it were not adjusted.

**Figure 4:**
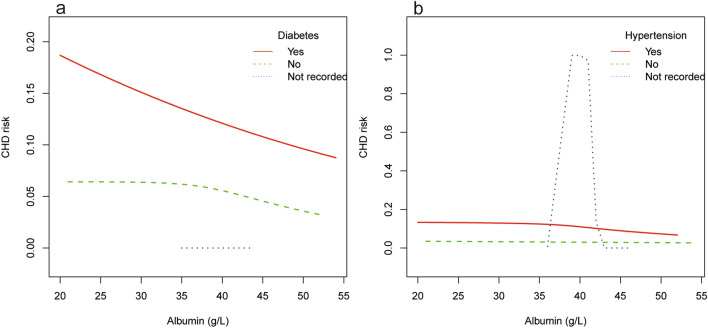
The relationship between serum albumin and coronary heart disease risk stratified by diabetes and hypertension. Adjust for: gender, age; race, diabetes, HDL-C, hypertension, LDL-C, creatinine. When diabetes or hypertension was the hierarchical variable, it was not adjusted.

Hierarchical smooth curve fitting showed that serum albumin and CHD risk showed an inverted U-shaped relationship in men (Fig. 3a), a nonlinear relationship in the participants under 60 years of age (Fig. 3b), and an approximate negative correlation independent of diabetes status (Fig. 4a). Furthermore, there was no significant correlation in individuals without hypertension (Fig. 4b).

## Discussion

This study analyzed data from the NHANES 2011–2018 database. After adjusting for confounding factors using multiple logistic regression, a negative correlation between serum albumin levels and CHD risk was found in people over 45 years of age, and the smooth curve fitting also showed the same results.

CHD is a serious disease that endangers human health. Determining CHD risk factors has always been a “hot” research topic. In the past, some studies have evaluated the relationship between serum albumin levels and the occurrence and development of CHD. Folsom et al. found that serum albumin was associated with risk factors for cardiovascular disease, without cardiovascular disease onset, which is inconsistent with our findings^11^. This difference may have been caused by too many confounding factors adjusted under the condition of a limited sample size. Other studies reported similar results to ours. Nelson et al. assessed a 5.2-year cohort study of the American population and found that low levels of serum albumin could increase the risk of CHD [HR = 1.18, 95% CI = (1.07, 1.30) ], which is consistent with our findings^13^. They also performed a stratified analysis and found that the result was statistically significant in women [HR = 1.30, 95% CI = (1.10, 1.53)]. However, we further performed stratified smooth curve fitting according to sex and found that in men serum albumin and CHD risk were inverted U-shaped. A 21.9-year follow-up study of the American population by Djoussé et al. found that low levels of serum albumin increase the incidence of myocardial infarction in both men [RR = 1.71, 95% CI = (1.17, 2.52)] and women [RR = 2.10, 95% CI = (1.10, 4.00)]^14^. As the most serious type of CHD, myocardial infarction was also related to serum albumin levels, which clinically significant. However, the definition of CHD in our study was broader. Therefore, a subgroup analysis of myocardial infarction could not be performed. Other studies found that serum albumin levels and cardiovascular disease risk, including CHD, such as angina pectoris and myocardial infarction, were also inter-related In Europeans^10,15^. Schalk et al. found a negative correlation between serum albumin and cardiovascular disease in a cohort study conducted in Amsterdam [RR = 0.88, 95% CI = (0.79, 0.98)]^15^. In a follow-up study of the general population in Copenhagen, Ronit et al. found that low levels of serum albumin increased the risk of ischemic heart disease [HR = 1.17, 95% CI = (1.08, 1.28)] and myocardial infarction [HR = 1.25, 95% CI = (1.09, 1.43)]^10^. A cross-sectional study in a Chinese Han population by Yang et al. also found that low serum albumin increased the risk of acute myocardial infarction [OR = 3.47, 95% CI = (2.86, 4.20)]^9^. Although the confounding factors adjusted in the study were not exactly the same and the magnitude of the effect value was also different, both our findings and the above-mentioned results could show that serum albumin was inversely correlated with CHD risk, and high levels of serum albumin may be a protective factor for CHD. The above findings have been verified by different research groups in the United States, Europe, and China. Our study further performed smooth curve-fitting based on stratified analysis, and found that the negative correlation between serum albumin and CHD risk was more pronounced in women, the participants over 60 years of age, and those with hypertension.

The mechanism by which serum albumin affect the pathogenesis of CHD may be related to its anti-inflammatory, anti-oxidative, and anti-platelet aggregation effects^16–18^. Oxidative stress is an important pathological process that is involved in the occurrence and development of coronary atherosclerosis^19^. Serum albumin is the most important antioxidant in the whole blood. It is rich in sulfhydryl groups, which can provide more than 80% of the total sulfhydryl groups in the plasma for scavenging reactive oxygen species^7^. In addition, serum albumin has a protective effect against vascular endothelial dysfunction caused by inflammation and oxidative stress during sepsis^20^. Albumin also has the effect of anti-platelet aggregation, it can prevent the platelet aggregation reaction induced by histones in a charge-dependent manner, and at the same time, it has the effect of anticoagulation^21,22^. The above-mentioned effects of serum albumin may represent the mechanism by which it was found related to CHD risk.

The main strength of this study was the reliability of the data. Data used in this study were collected from the NHANES database. This survey was characterized by rigorous design, reasonable sampling, large sample size and abundant variables. Second, in the process of data analysis, multiple regression analysis models were constructed by adjusting for different confounding factors. Meanwhile, independent variables were included in the models in different forms such as continuous and classified variables. In addition, this study performed smooth curve fitting to reflect the relationship between variables in a more intuitive image form. At the same time, this study also has several limitations. First, the design type of this study was a cross-sectional survey, Therefore, causality cannot be inferred. Therefore, further prospective research can be conducted based on the finding of this study. Second, this study was mainly carried out in the United States, and whether the conclusions can be applied to other countries and regions require further research. Since outcome indicators in this study were collected through self-report rather than objective measurement, there may be recall bias. Finally, although we adjusted for some confounding factors, some factors which have not been paid attention to, may still have a significant impact on the outcome indicators. Subsequent researchers can constantly improve the study design based on new findings.

In conclusion, our study found a negative correlation between serum albumin levels and the CHD risk. The mechanism behind this and whether it can reduce the risk of CHD by increasing the level of serum albumin in clinics still need to be further studied.

## Data Availability

The datasets obtained and analysed during the current study are available in the NHANES [https://www.cdc.gov/nchs/nhanes/index.htm].

## Abbreviations

CHD: Coronary heart disease
NHANES: National Health and Nutrition Examination Survey
PIR: Family income poverty ratio
BMI: Body mass index
HDL-C: High-density lipoprotein cholesterol
LDL-C: Low-density lipoprotein cholesterol
TG: Triglyceride
AST: Aspartate aminotransferase
WBC: White blood cell count
